# Cytokine production profile in intestinal mucosa of paediatric inflammatory bowel disease

**DOI:** 10.1371/journal.pone.0182313

**Published:** 2017-08-10

**Authors:** Serena Vitale, Caterina Strisciuglio, Laura Pisapia, Erasmo Miele, Pasquale Barba, Alessandra Vitale, Sabrina Cenni, Virginia Bassi, Mariantonia Maglio, Giovanna Del Pozzo, Riccardo Troncone, Annamaria Staiano, Carmen Gianfrani

**Affiliations:** 1 Institute of Protein Biochemistry, CNR, Naples, Italy; 2 Department of Woman, Child and General and Specialized Surgery, Second University of Naples, Naples, Italy; 3 Department of Translational Medical Science (Section of Paediatrics), and European Laboratory for the Investigation of Food-Induced Diseases, University Federico II, Naples, Italy; 4 Institute of Genetics and Biophysics Adriano Buzzati Traverso, CNR, Naples, Italy; Indiana University School of Medicine, UNITED STATES

## Abstract

In the recent years, the incidence of inflammatory bowel disease (IBD) has dramatically increased in young subjects, however, the pathogenesis of paediatric IBD is poorly investigated. In this study we aimed to evaluate the cytokine pattern and the phenotype of cytokine producing cells in the intestinal mucosa of paediatric patients affected by Crohn’s disease (CD) or ulcerative colitis (UC) and of non-IBD healthy controls (HC). Cytokine (IL-15, TNF-α, INF-γ) production was analyzed at basal condition and after mitogen stimulation either intracellularly by flow cytometry or in intestinal cell culture supernatants by enzyme-linked immunosorbent assay (ELISA). A higher frequency of enterocytes (EpCam+ cells) was observed in UC patients compared to CD or HC. An expansion of enterocytes producing IL-15 and TNF-α were found in IBD patients compared to HC. A marked expression of IL-15 in the intestinal epithelium of IBD patients was further confirmed by immunohistochemistry. Myeloid dendritic (CD11c+) cells producing TNF-α and INF-γ were increased in IBD biopsies. Unexpectedly, only after a strong mitogen stimulus, as phytohaemagglutinin, the frequency of CD3+ cells producing IFN-γ was increased in IBD compared to control intestinal mucosa. Interestingly, functional studies performed on organ cultures of intestinal biopsies with neutralizing anti-IL-15 monoclonal antibody showed a marked reduction of mononuclear cell activation, proliferation of crypt enterocytes, as well as a reduction of TNF-α release in organ culture supernatants. In conclusion, we found that in the gut mucosa of IBD children both enterocytes and dendritic cells produce proinflammatory cytokines. The over-expression of IL-15 by enterocytes in IBD intestine and the reduced IBD inflammation by IL-15 blockage suggests that this cytokine could be a therapeutic target in IBD.

## Introduction

Crohn’s disease (CD) and ulcerative colitis (UC) are chronic inflammatory bowel diseases (IBD) in which an abnormal immune response against the luminal microflora is thought to be the main causative factor [1,2]. Gut inflammation occurring in patients with IBD is characterized by the infiltration and activation of both adaptive branch, as T and B lymphocytes, and innate system, as macrophages and dendritic cells (DC), which in turn produce massive amounts of proinflammatory cytokines contributing to the typical mucosal lesions [3, 4]. It has been reported that cytokines released by T helper(Th)-1 cells, as interferon(IFN)-γ, tumor necrosis factor(TNF)-α and interleukin-12 (IL-12) are dominant in CD, whereas the Th2 cytokines, as IL-5, IL-9 and IL-13, are predominantly found in UC [5], though the role of IL-13 is still debated [6]. Furthermore, the great majority of the proinflammatory cytokines are produced by lamina propria mononuclear cells both in CD and UC [6], and very little is known regarding the epithelium compartment.

DC are the most potent professional antigen presenting cells, and in mucosal immunity they have an important role in maintaining the fragile equilibrium between tolerance and inflammatory response to mucosal antigens [7]. The involvement of mucosal DC in IBD pathogenesis has been also documented [8], though very little is known on their functions in paediatric IBD.

Enterocytes have a pivotal role in maintaining the integrity of intestinal mucosa, where they guarantee gut homeostasis by sampling luminal agents through several receptors, such as the pathogen recognition receptors (PPRs) expressed on their surface [4, 9]. Given the prominent role of enterocytes in the intestinal immune homeostasis, dysfunctions within the epithelial layer can be associated with IBD pathogenesis. Interestingly, very recent evidences underlined an active role of enterocytes as nonimmune inflammatory cells in the IBD mucosal lesions [10].

IL-15 is a pleiotropic cytokine that is expressed on the surface of monocyte, macrophages, dendritic cells and intestinal epithelial cells, where is found markedly expressed [11] in response to inflammatory stimuli. IL-15 is involved in several inflammatory mechanisms mediated by both adaptive and innate immune systems [12, 13, 14], particularly in the gluten-dependent mucosal lesion, where it is a key mediator of the epithelial layer changes [15]. IL-15 has various functions due to complex signal transduction pathways activated after the ligation of either a specific receptor (IL-15Rα), or the βchain of IL-2 receptor [16]. IL-15 is an important growth and activator factor for mucosal intraepithelial lymphocytes [17], and it has also a marked anti-apoptotic function, as previously shown [18].Quite recently, it has been demonstrated a higher expression of IL-15 in IBD colonic mucosa, thus suggesting a role of this cytokine in the IBD pathogenesis [19, 20].

The contribution of the adaptive T cell-mediated response against luminal components in IBD intestinal lesions has been observed in many studies [21, 22]. Particularly, the role of cytokines released by bacterial-primed T cells is undoubted, and several therapeutic approaches to block cytokine-mediated inflammatory cascades are currently under investigation [23]. While it has been proved the crucial function of T cells in IBD, many aspects of the inflammatory process in IBD mucosa still need to be clarified, in order to develop more appropriate and disease-specific therapeutic strategies. A deeper dissection of the cross-talk between the different mucosal compartments, such as enterocytes, dendritic cells and T lymphocytes, especially with regard to the cytokines mediating the interaction of both innate and adaptive immune cells, will represent a step forward in the knowledge of IBD intestinal inflammation [4].

Though a great progress was reached in the comprehension of the immune mechanisms responsible of gut inflammation in adult subjects with IBD, very little is known on both the etiology and inflammatory cascade leading to these chronic gut disorders in subjects of paediatric age. We believe that the marked rising in the incidence of IBD in childhood, renders of particular interest such studies. Indeed, the distinction between adult and paediatric IBD is a current topic, as children with IBD seem to be a distinctive population with specific peculiarities requiring a highly skilled and specialized approach for diagnosis and treatment [24]. Paediatric disease onset is often more severe and aggressive [25] and it represents a privileged access for the study of pathogenetic mechanisms, since it lacks numerous confounding factors, that complicate the analysis in adult patients. Therefore, the aim of our study was to evaluate in paediatric patients with IBD the cytokine production profile and the activation status of both lamina propria and epithelial cells compartments.

## Materials and methods

### Study population

The study population included a total of 71 young subjects divided into 3 groups on the basis of the clinical diagnosis: 26 (mean age 14.6 yr; range 7.8–18 yr) were affected by CD, 26 (mean age 13.9 yr; range 8.6–18.4 yr) affected by UC, and 19 (mean age 10.4 yr; range 3.4–18.5 yr) were non-IBD controls (HC). HC were subjects who underwent ileo-colonoscopy to either exclude an organic disease or to practice polypectomy, and who did not present any signs of mucosal inflammation or disease, except for some patients that had single and isolated juvenile polyps. For the IBD diagnosis, all children underwent ileo-colonoscopy, upper GI endoscopy and imaging studies, including abdominal ultrasound and entero-MRI, or a small bowel follow-through. The diagnosis of CD and UC was based upon conventional clinical, radiological and endoscopic features and was confirmed by histopathological examination of inflamed areas of intestinal biopsies [26]. Demographic and clinical characteristics of enrolled subjects are described in **Table 1**.

**Table 1:** Clinical features of paediatric subjects enrolled.

	CD	UC	HC
Patients	26	26	19
Gender, male/female	14/12	14/12	13/6
Mean age (year and range)	14.6 (7.8–18)	13.9 (8.6–18.4)	10.4 (3.4–18.5)
Mean age at diagnosis (year and range)	11.2 (2.9–18)	11.6 (5–15.8)	na
Mean disease duration (month and range)	2.30 (0–6.2)	2.6 (0–11.5)	na
Therapy:			na
Aminosalicylates	7	10	
Immunosuppressants	5	10	
Aminosalicylates and immunosuppressants	5	1	
Monoclonalantibodies	1	0	
Nutritional	2	0	
No therapy	6	5	

na indicates not applicable

### Intestinal cell isolation and stimulation

Intestinal biopsies processed in this study were taken only from macroscopically uninflamed areas of ileum and colon traits, as the biopsies taken from the inflamed area were all used for diagnostic and histopathological analysis. Intestinal cells were isolated from the biopsies according to a well-established procedure, as previously described [27, 28]. Briefly, mucosal samples were washed in saline solution and digested with collagenase A from *Clostridium hystolyticum* (1mg/mL; Roche, Mannheim, Germany) in 2 mL of culture medium, RPMI-1640, supplemented with 1% penicillin/streptomycin antibiotics (Lonza Group Ltd, Basel, Switzerland) for 1 hour and 30 minutes at 37°C and 5% CO_2_, stirring the plate every 15 minutes. The cellular suspension was then passed through a 40-μm cell strainer filter (BD Falcon, Durham, USA), before the measurements of cell viability and recovery. The median value of intestinal cells isolated from 4–5 biopsies was 5.7x10^6^ cells, and the range was 1.8-13x10^6^cells.

In the experiments assessing the intracytoplasmic cytokine production, intestinal cells (from 13 CD, 12 UC and 11 HC individuals) were cultured at a cellular density of 1x10^6^-1.4x10^6^ cells in 24-wells plates (Sarstedt; Newton, NC, USA) in complete medium (RPMI-1640 with 10% fetal calf serum and supplements). Because of the long experimental procedures, the intestinal cells were cultivated 16 hours with 100 ng/mL GM-CSF and 50U/mL of IL-2 (both from R&D System, Minneapolis, MN, USA), in order to maintain alive the cells until the cytokine analysis. To measure the spontaneous cytokine production (in absence of mitogen stimulation), brefeldin A (5 μg/ml; Sigma-Aldrich) was added to cell cultures, whilst the mitogen-induced cytokine production was analyzed by adding a mixture of phorbol 12-myristate 13-acetate (stock 40.5 μM), ionomycin (stock 670 μM), brefeldin A (stock 5.3 mM), monesin (stock 1mM) at the final dilution 1:500, as indicated by the manufacturers’ instruction (Cell Stimulation Cocktail, eBioscience Inc. SanDiego, CA), for 5 hours. To evaluate the cytokine production by DC upon bacterial stimulation, we incubated intestinal cells with bacterial (*E*.*coli*) particles (20 mg/mL of Alexa-488-labeled *E*. *coli* bioparticles, Invitrogen, Carlsbad, CA), for 2 hours. Thereafter, both stimulated and unstimulated cells were harvested and treated for flow cytometry analysis, as described below.

In the experiments performed to measure cytokine released in the culture supernatants, intestinal cells (2x10^5^ cells/well), obtained from 8 CD, 7 UC and 8 HC, were plated in triplicates in 96-wells plates (BD Bioscience, Oxford, UK) with medium alone or stimulated with phytohaemagglutinin (PHA, 2 μg/ml; Sigma-Aldrich, Stockholm, Sweden). After 48 hours of incubation, cell supernatants were collected and stored at -20°C until the ELISA assay was performed.

### Flow cytometry

Intestinal cells were stained with following fluorochrome-labeled monoclonal antibodies: CD3-PerCP-Vio700, CD11c-PE, HLA-DR-PerCP, CD326 (EpCam)-APC, HLA ClassI-PE-Cy5, IL-15-PE. Intracellular cytokine staining was performed with the fluorochrome-conjugated monoclonal antibodies anti-IFN-γ-APC and anti-TNF-α-APC/Cy7. Appropriate isotype-matched control monoclonal antibodies were included in all staining experiments. All antibodies were purchased from BD Biosciences (San Jose, CA), or Miltenyi Biotec (Bologna, Italy), and used at concentration according to the manufacturer’s instructions. At least 1x10^5^ viable cells (assessed at microscope by trypan blue dye exclusion) were used for each staining done in phosphate saline (PBS)/0.5% bovine serum albumin (BSA) buffer. Surface-staining of cells was carried out at 4°C for 30 minutes; then the cells were fixed with 2% paraformaldehyde and intracellular staining was performed in permeabilization buffer (PBS/BSA 0.5% with 0.5% saponin). All multi-color flow cytometry analyses included the appropriate fluorescence-minus-one control (FMOC) sample. Samples were acquired with FACSCanto II (BD Biosciences) equipped with two lasers, that permits the simultaneous analysis of up to 5 different fluorescence and 2 physical parameters. The results were analyzed with FlowJo software (MiltenyiBiotec). All analyses of cytokine-stained cells were done within a gate based on the forward-scatter/side-scatter characteristics that excluded dead cells assessed by propidium iodide staining running in parallel.

### Cytokine quantification in intestinal cell culture supernatants

The concentrations of TNF-α, IFN-γ, and IL-17 were analyzed in cell supernatants collected after 48 hours of incubation with medium alone or with the indicated mitogen. The cytokines production was measured by ELISA using commercially available kits provided by Mabtech, (Nacka Strand, Sweden), according to the manufacturer’s instructions. The sensitivity of ELISA kits was 12 pg/mL for TNF-α, 2 pg/mL for IFN-γ, and 2 pg/mL for IL-17.

### Organ culture of ileocolonic biopsies

During ileo-colonoscopy, at least 3 biopsies were taken respectively from the rectum for UC patients (n = 7) and from the ileum for CD patients (n = 5). Mucosal specimens were used to perform organ culture experiments and placed on a stainless steel mesh positioned over the central well of an organ culture dish (Becton Dickinson, New York, USA) with the mucosal surface of the biopsies oriented on the top of the well. The biopsy specimens were cultured for 24 hours at 37° in the presence of medium alone or anti-IL-15 monoclonal antibody (5μg/ml; R&D Systems, Minneapolis, MN). The medium was composed from RPMI 1640 (80%; Sigma, Milan, Italy) supplemented with fetal bovine serum (15%; Life Technologies-GibcoBRL, Milan, Italy), L-glutamine (2mM; Life Technologies-GibcoBRL), penicillin (100 U/ml), streptomycin (100 μg/ml) (Life Technologies-GibcoBRL), and insulin (1mg/ml; Sigma). After 24 hours of culture, the tissues were embedded in OCT and processed for immunohistochemistry. The supernatants were collected and stored at -20°C until ELISA assay was performed.

### Immunohistochemistry

Immediately afterwards the ileo-colonoscopy, one specimen was embedded in an optimal cutting temperature compound (OCT; Killik, Bio-Optica, Milan, Italy) and stored in liquid nitrogen until used. IL-15 expression was assessed on the surface epithelium of intestinal fragments in basal condition, as previously reported [29]. The IL15 staining on epithelial cells was graded as: no signal, weak, moderate, or strong.

After 24h incubation with anti-IL15 antibody, immunohistochemical staining for CD25 positive cells was performed using four-μm frozen ileum and rectal sections, as previously reported [30]. The density of cells expressing CD25 in the lamina propria was evaluated within a total area of 1 mm^2^.

Proliferation of crypt epithelial cells was evaluated in cultured intestinal biopsies by Ki67 antigen detection, as previously reported [29]. The percentage of Ki67 positive cells was calculated dividing the number of Ki67 positive cells in the crypt by the total number of crypt enterocytes.

All sections were evaluated with a light microscope Axioskop2 plus (Zeiss).

### Cytokine quantification in organ culture supernatants

TNF-α and IFN-γ were measured in organ culture supernatants collected after 24 hours of incubation with medium alone or with anti-IL-15 antibody by ELISA using commercially available kits provided by Mabtech, (Nacka Strand, Sweden), according to the manufacturer’s instructions as stated above.

### Statistical analysis

Statistical analyses for flow cytometry analysis and ELISA assay were performed using SPSS software package for Windows (version 13.0; SPSS, Chicago, IL). The Mann Whitney U test/Wilcoxon test for normally distributed variables was used where appropriate. Statistical analyses for immunohistochemistry experiments were performed using GraphPad Prism 4 for Windows, version 4.03. Data from organ culture experiments were compared by paired t test. A p value < 0.05 was considered statistical significant.

### Ethical considerations

The Institutional Review Board of the University of Naples “Federico II” approved the study protocol and questionnaire with the registration number 107/15. Informed consent was obtained from parents of all children, and from children themselves if older than 13 years.

## Results

### Cytokine production profile in intestinal IBD mucosa

By an *ex vivo* flow cytometry approach, we have analyzed the frequency of intestinal mucosal cells that spontaneously produced cytokines in uninflamed area of young patients with IBD, or age matched controls. We detected the frequency of cells that produced IL-15, IFN-γ or TNF-α in colonic biopsies from 13 CD, 12 UC, and 11 HC, without further *in vitro* stimulation with mitogen.

**Fig 1:**
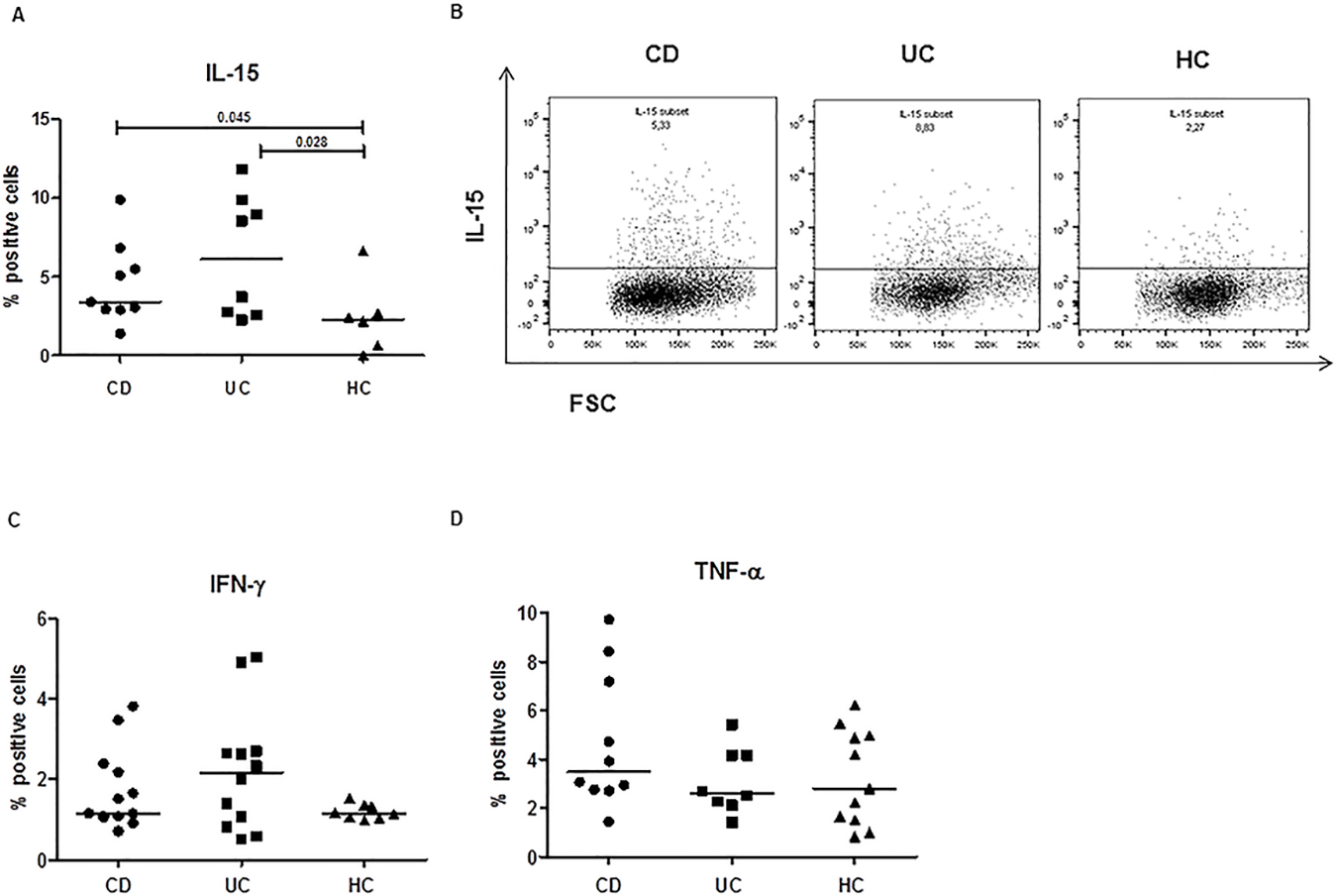
Increased frequencies of IL-15 producing cells in the intestinal mucosa of paediatric patients with IBD. The frequency of cells spontaneously producing IL-15, TNF-α or IFN-γ was analysed in uninflamed areas of intestinal biopsies from paediatric patients with Crohn’s disease (CD), ulcerative colitis (UC), and non-IBD healthy controls (HC). Cytokine production was assessed by multi-color flow cytometry performed on intestinal cells, after an overnight incubation with IL-2 and after additional 5 hours with the Golgi transport inhibitor BFA. The mucosal infiltration of IL-15 expressing cells was evaluated by surface staining, whilst TNF-α and IFN-γ producing cells were detected by intracytoplasmic staining. (**A**) Overall percentage of IL-15-positive cells and (**B**) one representative flow cytometry dot plot of IL-15 stained cells from each group of patients are shown. Numbers correspond to the cytokine positive cells gated on forward- and side-scatter properties to exclude dead cells, debris and granular cells. The frequencies of intestinal cells producing TNF-α (**C**) and IFN-γ (**D**), evaluated by intracellular staining, are shown. Each point represents the percentage of positive cells in pooled intestinal mucosa biopsies taken from one single subject. Horizontal bars are the median values. The Mann-Whitney U test was applied to evaluate statistical significant differences among the three groups.

In particular, the evaluation of IL-15-positive cells was determined by a membrane surface staining, whilst the number of IFN-γ and TNF-α positive cells was assessed by intracellular staining with specific monoclonal antibodies. The percentage of IL-15-cells resulted significantly higher in the mucosa of CD and UC patients compared to non-IBD controls (CD: median 3.41, range 1.4–9.9%; UC: 6.14, 2.3–11.8%;HC: 2.3, 0–6.6%, p<0.03,UC vs HC; p<0.05, CD vs HC), **Fig 1A and 1B**. An increase of cells producing IFN-γ was observed in UC, though no statistical significant differences were found compared to the other two groups, **Fig 1C**. Surprisingly, when we looked at the intracellular TNF-α positive cells, no marked differences were observed between the IBD and the control group, **Fig 1D.**

### Levels of TNF-α and IFN-γ secreted by intestinal mucosa cells

**Fig 2:**
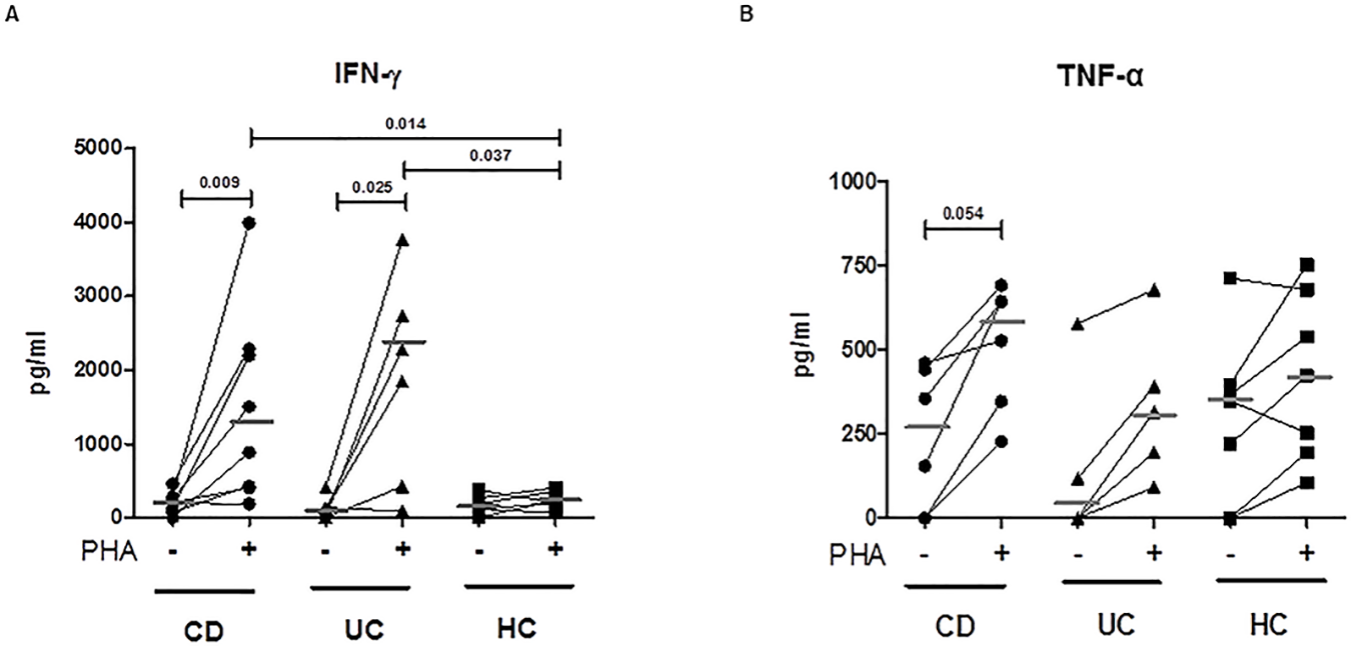
Intestinal mucosa cells from young patients with IBD secrete IFN-γ and TNF-α after a brief mitogen stimulation. (**A**) IFN-γ and (**B**) TNF-α were measured by ELISA in the culture supernatants of intestinal cells isolated from intestinal mucosa of CD and UC patients, and from non-IBD controls (HC). Soon after the isolation from mucosal tissues, 2x10^5^ cells were stimulated for 48 hours with medium or with phytohaemagglutinin (PHA, 2 μg/ml) in triplicates. Each line connects the cytokine produced by unstimulated to PHA stimulated cells, from each single subject. Horizontal bars are the median values. The Mann-Whitney U/Wilcoxon test was applied, as appropriate to evaluate statistical significant differences.

To further investigate the cytokine production profile in intestinal mucosa of IBD paediatric patients, we measured by ELISA the IFN-γ, TNF-α, and IL-17 secreted by intestinal cells either at basal condition (unstimulated) or after a mitogen (PHA) stimulation. In agreement with FACS analysis, we found no differences in the levels of IFN-γ in unstimulated conditions among the three groups (CD: median 204.4, range 0–285 pg/mL; UC: 101.6, 0–406.3 pg/mL; HC: 151.1, 0–376.3 pg/mL, p = ns). Notably, upon the activation with PHA a marked IFN-γ production was detected in cell supernatants from IBD patients (CD: median 1194.8, range 188–3991.2 pg/mL; UC: 2062.4, 89.7–3767.5 pg/mL, p<0.01, CD unstimulated vs CD stimulated; p<0.03, UC unstimulated vs UC stimulated), whilst the IFN-γ level measured in cultures from HC intestinal mucosa (HC: 221.8,65.8–405.1 pg/mL) resulted much lower compared to IBD mucosa (p<0.02, CD vs HC; p<0.04, UC vs HC), **Fig 2A**. A sustained production of TNF-α upon PHA stimulation was observed in both CD and UC mucosal cells (CD: median 584.5, range 346.7–691.2 pg/mL; UC: 316, 91.7–677.8 pg/mL) compared to unstimulated cells (CD: median 255.2, range 0–461.2 pg/mL; UC: 58.7, 0–577.8 pg/mL), though the statistical significance was only reached in CD intestinal cells (p = 0.05). Unexpectedly, high levels of TNF-α were found in cell supernatants from HC biopsies despite the experimental conditions (median 348.4, range 0–712 pg/mL; 423.4, 105.3–751.7 pg/mL, respectively at baseline or PHA-stimulated), **Fig 2B**. Instead, the production of IL-17 was very low or undetectable, either at basal level and after PHA stimulation. IL-15 was not measured in cell supernatants, since it has been reported that this cytokine is biologically active when bound to its specific receptor on the cell membrane surface [31, 12, 32].

### Phenotype of inflammatory cytokine producing cells in colonic mucosa

To further dissect the phenotype and the activation status of various cell types producing cytokines in intestinal mucosa of young patients with IBD, we performed additional *ex vivo* flow cytometry analysis. By using specific cell markers, we investigated enterocytes identified as EpCam (epithelial cell adhesion molecule) positive cells, myeloid dendritic cells identified as CD11c positive cells, and T lymphocytes identified as CD3 positive cells. More specifically, we performed the following fluorochrome multicolor staining combinations: i) EpCam, IL-15, TNF-α, HLA Class I; ii) CD11c, HLA DR, TNF-α, IFN-γ; iii) CD3, TNF-α, IFN-γ.

#### i) Enterocytes

**Fig 3:**
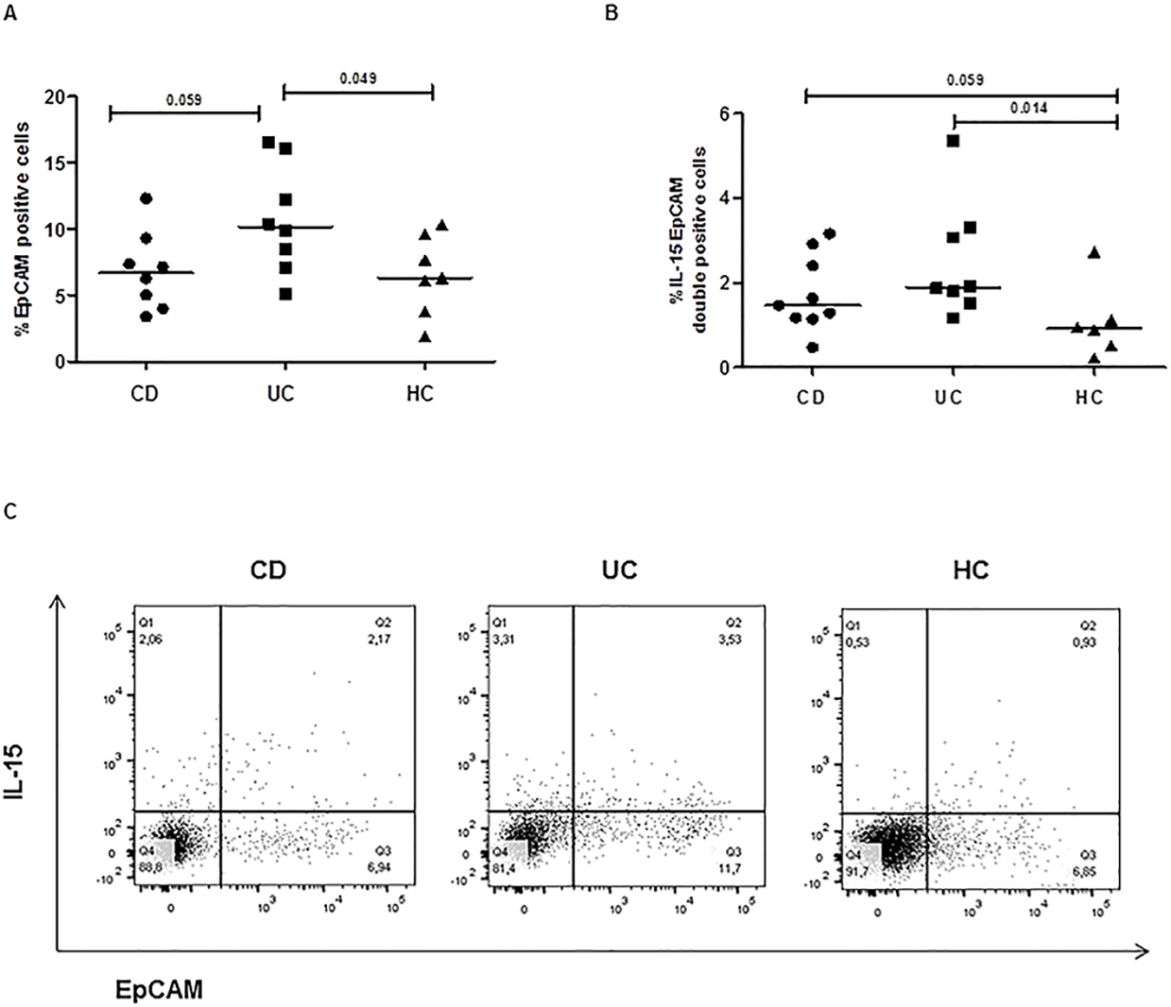
Expansion of enterocytes expressing IL-15 in intestinal mucosa of paediatric patients with IBD. Enterocytes were identified as EpCam positive cells. (**A**) The percentage of EpCam positive cells, and (**B**) IL-15 and EpCam double positive cells were evaluated in freshly isolated intestinal cells, at basal condition without *in vitro* mitogen stimulation. Each point represents the percentage of positive cells from one single subject.(**C**) Flowcytometry dot plots of IL-15 and EpCam stained cells are reported from one representative subject of each group (CD, UC, HC). Horizontal bars are the median values. The Mann-Whitney U test was applied to evaluate statistical significant differences.

We first assessed the level of enterocyte expansion in uninflamed mucosal tissues, by detecting the percentage of EpCam+ cells. We found that the proportion of EpCam+ cells was significantly higher in UC compared to the healthy mucosa (UC: median 10.02, range 5.1–16.5%; HC: 6.3, 1.9–10.3%, p<0.05). Interestingly, the number of EpCam stained cells isolated from UC mucosa resulted also greater than the level found in CD mucosa (CD: median 6.7, range 3.4–12.3%, p<0.06), **Fig 3A**. Several studies have reported that enterocytes are an important intestinal source of inflammatory IL-15, both in healthy and in food related disorders, as celiac disease [33, 34]. We next evaluated whether the enhanced levels of IL-15 expression we found in IBD mucosa (**Fig 1A and 1B**) could be specifically produced by enterocytes. Interestingly, EpCam and IL-15 double positive cells were increased in intestinal samples from IBD patients, and particularly from those with UC (CD: median 1.5, range 0.5–3.2%; UC: 1.9, 1.2–5.4%; HC: 0.9, 0.2–2.7%; p<0.02, UC vs HC and p<0.06, CD vs HC), **Fig 3B and 3C**.

**Fig 4:**
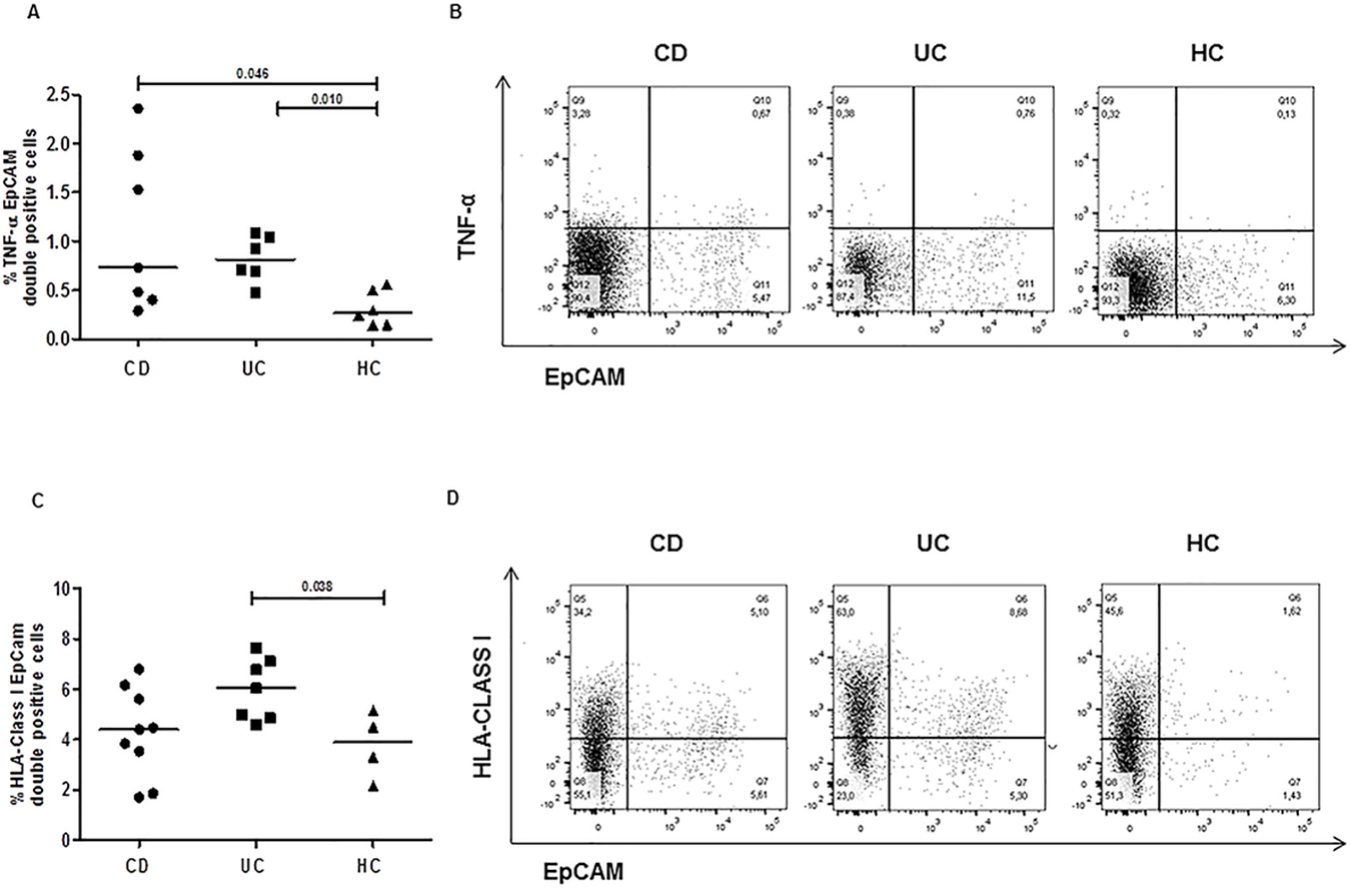
Gut mucosa of paediatric patients with IBD has an increased frequencies of enterocytes producing TNF-α and expressing HLA Class I molecules. Enterocytes (EpCam positive cells) that spontaneously produced TNF-α and expressed HLA Class I were evaluated in intestinal biopsies from young subjects with Crohn’s disease (CD), ulcerative colitis (UC), and non-IBD healthy controls (HC). Cells were collected and stained as described in Fig 1. Overall percentage and representative dot plots of EpCam and TNF-α (**A and B**),and of EpCam and HLA-Class I (**C and D**) double positive cells in pooled intestinal biopsies from each group are shown. Horizontal bars are the median values. The Mann-Whitney U test was applied to evaluate statistical significant differences.

Because it has been shown that the intestinal epithelial cells are able to produce TNF-α [35], we next looked at TNF-α production in the EpCam+ cells subset. An increased frequency of EpCam+ cells producing TNF-α was found in IBD mucosa compared to healthy colonic tissues (CD: median 0.7, range 0.3–2.4%; UC: 0.8, 0.5–1.1%; HC: 0.3, 0.1–0.61%; p<0.02, UC vs HC and p<0.05, CD vs HC), indicating that enterocytes are a source of proinflammatory TNF-α in colonic tissues of young patients with IBD, **Fig 4A and 4B**.

Next, we looked at the HLA (human leukocyte antigen) Class I expression on enterocytes, as this molecule is constitutively expressed on almost all cells either of immune or nonimmune systems, but strongly upregulated in response to infection or stress signals [10, 36]. Consistently with above findings (**Fig 3A**), UC mucosa displayed an increased number of HLA Class I-expressing enterocytes in comparison to the healthy mucosa (UC: median 6, range 4.6–7.7%; HC: 3.9%, 2.1–5.1%, p<0.04), **Fig 4C and 4D.**

#### ii) Myeloid dendritic cells

**Fig 5:**
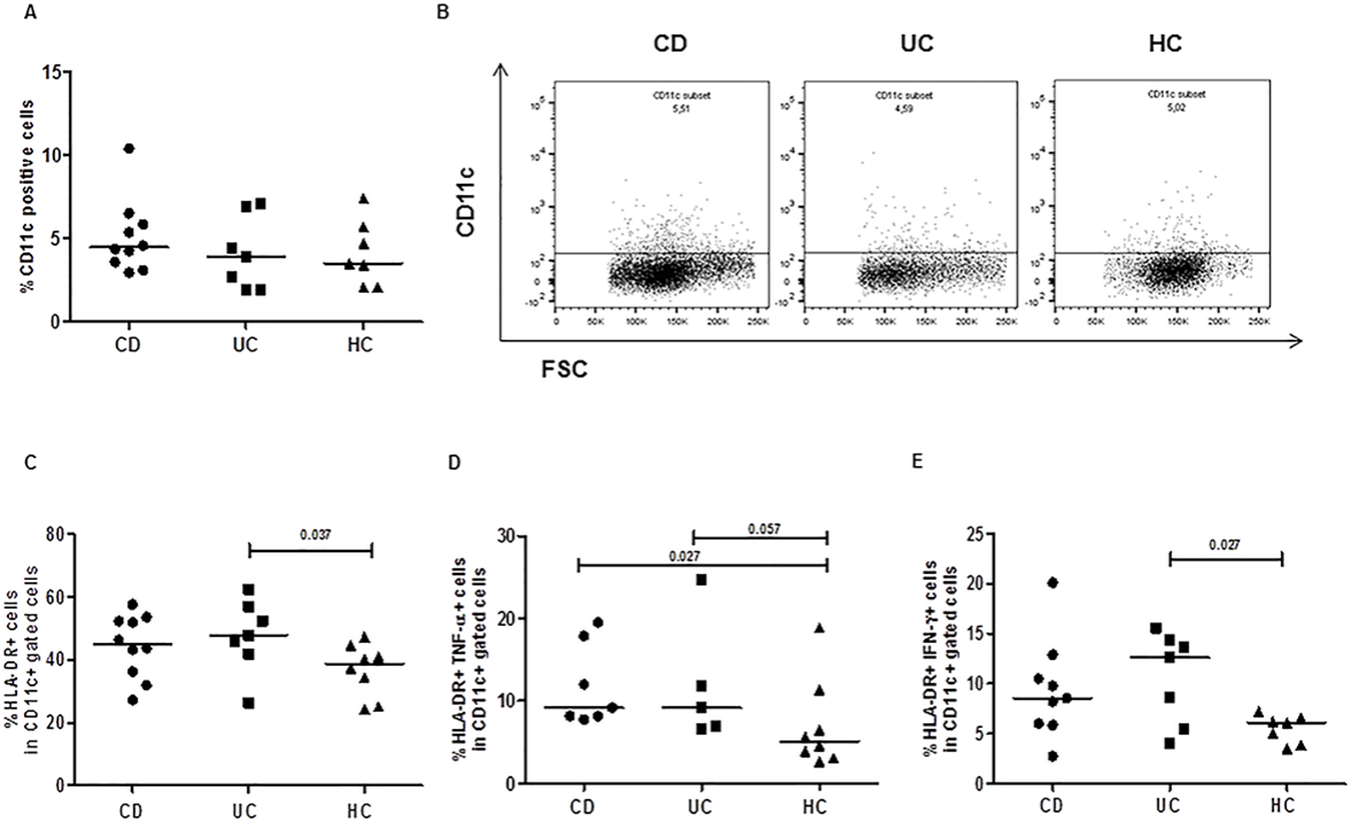
Dendritic cells infiltrating the intestinal mucosa of young patients with IBD produce TNF-α and IFN-γ. Intestinal dendritic cells (DC) were evaluated as CD11c positive cells. Overall frequencies and representative dot plots of CD11c positive cells detected in intestinal mucosa from Crohn’s disease (CD), ulcerative colitis (UC), and non-IBD controls are shown. (**A and B**) The frequencies of CD11c positive cells expressing the HLA DR activation marker (**C**), and producing TNF-α (**D**),or IFN-γ (**E**) are shown. All data are referred to the cell infiltration observed in intestinal biopsies at basal, unstimulated condition. Horizontal bars are the median values. The Mann-Whitney U test was applied to evaluate statistical significant differences.

The majority of studies addressing the phenotype and function of mucosal DC have used the CD11c expression as specific surface marker of myeloid dendritic cell lineage [37], though macrophages, monocytes, neutrophils, and some B cells may express this molecule [38]. We found similar percentages of CD11c+ dendritic cells (CD: median 4.5, range 3.1–10.4%; UC: 3.9, 1.9–7.1%; HC: 3.5, 2.1–7.4%, p = ns) in mucosal samples from the IBD and control mucosa, **Fig 5A and 5B**. Nevertheless, when we focused the analysis to the specific region of CD11c positive dendritic cell subset, we observed that DC were more activated in IBD than in healthy mucosa, as they expressed the activation marker HLA-DR, as well as the proinflammatory cytokines TNF-α and IFN-γ. More specifically, we found within the CD11c+ subset a higher number of cells expressing HLA-DR in IBD mucosa than in control mucosa at the basal condition (CD: median 45, range 27.2–57.5%; UC: 47.7, 26.3–62.4%; HC: 38.7, 24.3–47.2%) with a statistical significant increment in UC versus HC (p<0.04), **Fig 5C,** as well as an enhanced frequency of activated cells producing TNF-α (CD: median 9.2, range 7.8–19.5%; UC: 9.2, 6.7–24.8%; HC: 5.1, 2.6–18.9%; p<0.03, CD vs HC and p<0.06, UC vs HC) and IFN-γ (CD: median 8.5, range 2.7–20.1%; UC: 12.6, 4.1–15.6%; HC: 6.1, 3.5–7.2%; p<0.03, UC vs HC), **Fig 5D and 5E.** Notably, these findings are in accordance with our previous report on peripheral blood monocyte-derived DC, showing a proinflammatory phenotype of myeloid dendritic cells in paediatric IBD [39]. We also investigated the cytokine production by CD11c+ DC upon a brief stimulation with *E*.*coli* bioparticles. We did not observe statistical significant differences after the bacterial incubation, except for the DC derived from UC mucosa that showed an increased percentage of CD11c+ cells expressing HLA-DR and producing TNF-α (**S1 Fig).**

#### iii) T cells

**Fig 6:**
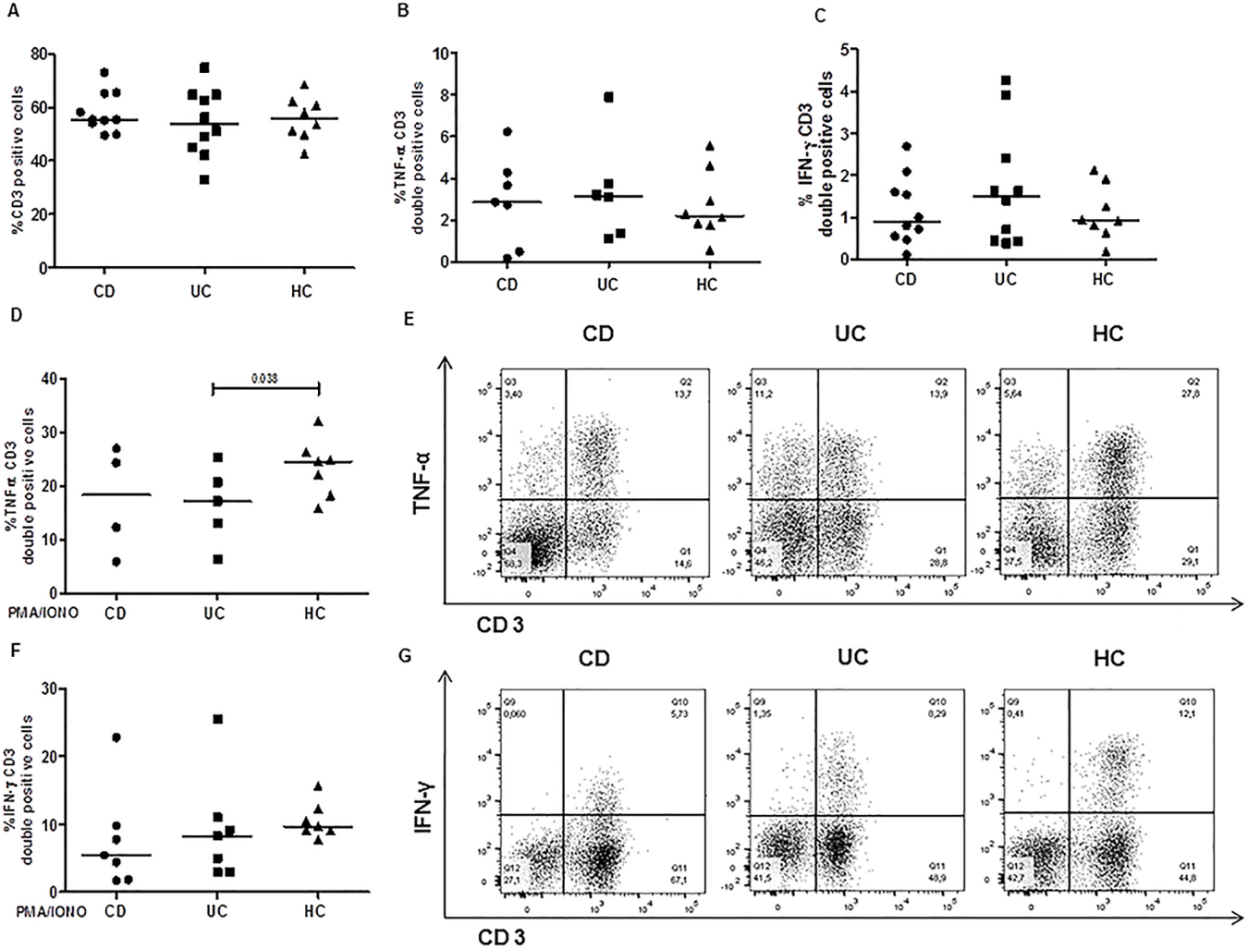
Healthy intestinal mucosa contains T lymphocytes prone to secrete proinflammatory mediators only upon a strong *in vitro* stimuli. T lymphocytes (CD3 positive cells) producing cytokines were analyzed in intestinal biopsies of IBD and control paediatric subjects either at basal condition or after a brief *in vitro* mitogen stimulation. (**A**) Unstimulated CD3 positive cells infiltration and proportions of CD3 positive cells spontaneously producing TNF-α (**B**), and IFN-γ (**C**) in CD, UC and control biopsies are shown. Frequencies of CD3 positive cells producing TNF-α (**D and E**) and IFN-γ (**F and G**) after stimulation for 5 hours with PMA/Ionomycin are illustrated. Horizontal bars are the median values. The Mann-Whitney U test was applied to evaluate statistical significant differences.

Finally, we expanded the *ex vivo* analysis to T (CD3+) cells. As observed in DC subset, we did not find differences in the percentage of T lymphocytes in mucosal samples among the three paediatric groups (CD: median 55.4, range 49.6–73%; UC: 53.7, 33.1–74.8%; HC: 55.7, 42.6–68.5%), **Fig 6A**. Similarly, no differences in the frequency of CD3+ cells spontaneously producing TNF-α (CD: median 2.87, range 0.2–6.2%; UC: 3.2, 1.1–7.9%; HC: 2.2, 0.5–5.6%), and IFN-γ (CD: median 0.9, range 0.1–2.7%; UC: 1.5, 0.4–4.3%; HC: 0.9, 0.2–2.1%), were detected among the three groups (**Fig 6B and 6C)**.

To further analyze the production of these two inflammatory cytokines in T lymphocytes infiltrating the paediatric IBD mucosa, we did additional experiments examining the cytokine production in CD3+ cells induced by a strong mitogen, such as PMA/Ion. Unexpectedly, we observed an increased percentage of CD3+ cells positive for either TNF-α (**Fig 6D and 6E**) or IFN-γ (**Fig 6F and 6G**) in control mucosa, with a significant difference in the frequency of CD3 TNF-α double positive cells between HC and UC patients (CD3+TNF-α+, CD: median 18.4, range 6.0–27%; UC: 17.2, 6.5–25.4%; HC: 24.6, 15.9–32.1%, p<0.04, HC vs UC; CD3+IFN-γ+, CD: 5.4, 1.7–22.8%; UC: 8.2, 2.9–25.5%; HC: 9.6, 7.7–15.7%, p = ns).

### IL-15 expression on the surface epithelium in IBD intestinal mucosa

We first have evaluated the IL-15 expression in the intestinal mucosa of IBD patients. In particular, we investigated by immunohistochemistry the basal IL-15 expression in the epithelium of ileum or rectal biopsies taken from 5 CD and 7 UC subjects. IL-15 resulted expressed on the surface of intestinal epithelium in both CD and UC patients with a distribution pattern in some cases patchy. Overall, the epithelium expression of IL-15 was heterogeneous, ranging in the majority of cases from moderate to weak intensity (**Table 2**).

**Table 2:** IL-15 epithelial expression in the intestinal biopsies from paediatric IBD patients.

Patients	Age (yr,m)/gender	Age at diagnosis (yr,m)	Therapy	IL-15 expression
**CD#1**	17.6/M	9	Nutritional	moderate/strong
**CD#2**	16.6/F	13.6	Aminosalicylates	moderate
**CD#3**	15.4/F	13.4	Nutritional	moderate
**CD#4**	17.3/M	11.5	Aminosalicylates	weak/moderate
**CD#5**	13.9/M	10.2	Immunosuppresants/ Aminosalicylates	weak/moderate
**UC#1**	10.4/F	9	Immunosuppresants	moderate/strong
**UC#2**	16.5/M	14.8	Aminosalicylates	weak/moderate
**UC#3**	17.7/M	12	Aminosalicylates	moderate
**UC#4**	14.4/M	12.6	Aminosalicylates	weak/moderate
**UC#5**	13.9/M	12.1	Immunosuppresants	moderate
**UC#6**	12/F	6	Immunosuppresants/ Aminosalicylates	moderate
**UC#7**	12/F	14.9	Immunosuppresants	weak/moderate

### Effect of IL-15 neutralization on organ culture of IBD intestinal mucosa

**Fig 7:**
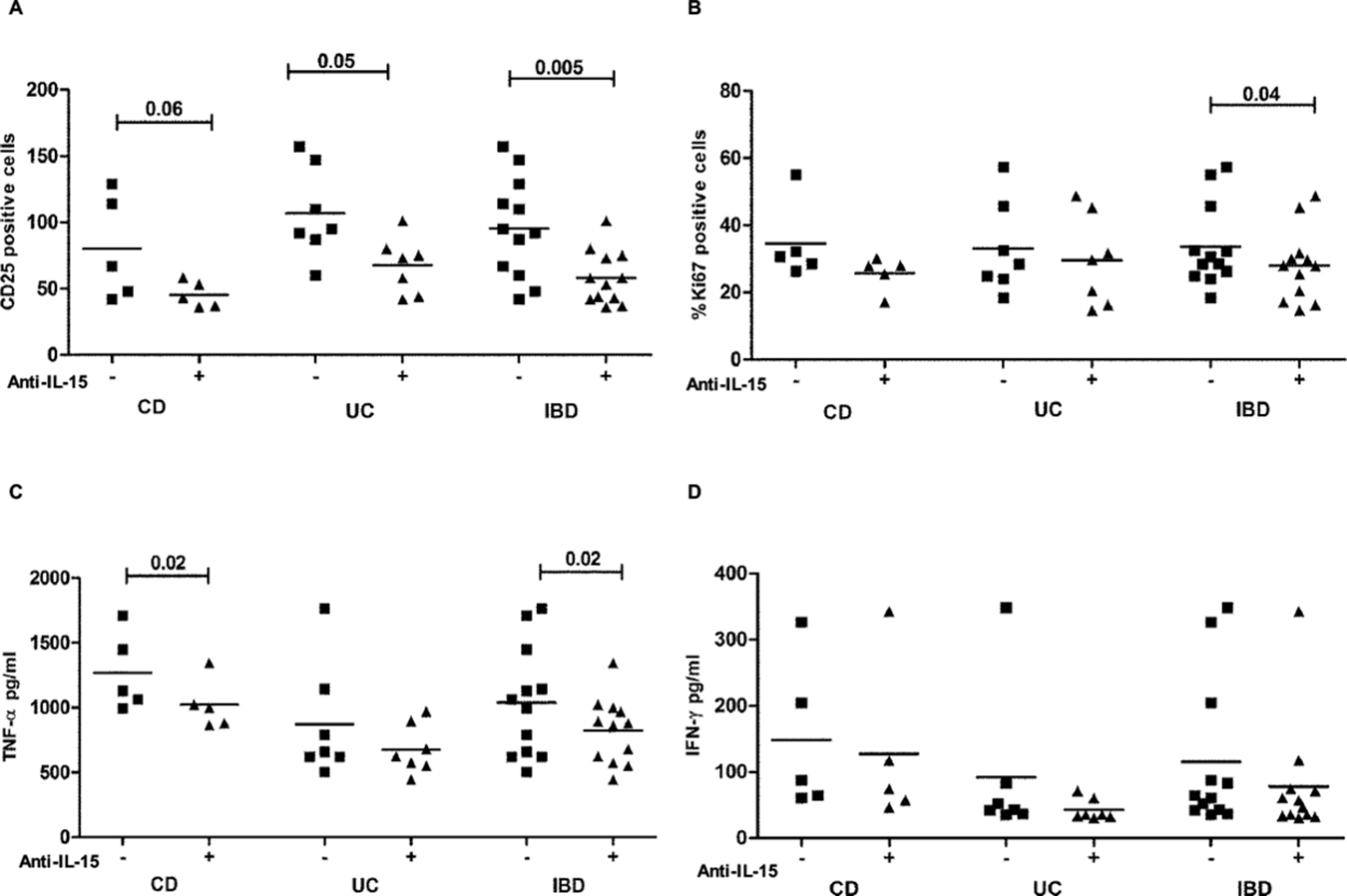
IL-15 blocking reduces inflammation in organ culture of intestinal mucosa from young patients with IBD. The organ culture experiments were performed on colonic or rectal fragments from CD (n = 5) and UC patients (n = 7), respectively. After 24 hours of culture with anti-IL-15 antibody or medium alone, the tissues were processed for immunohistochemistry (**A and B**) whereas the supernatants were collected to detect cytokines by ELISA (**C and D**). (**A**) The number of activated (CD25 positive) mononuclear cells in lamina propria of CD, UC and overall IBD patients is shown. The density of cells expressing CD25 was evaluated within a total area of 1 mm^2^ of lamina propria. (**B**) The percentage of proliferating crypt epithelial cells (Ki67 positive cells) is illustrated. The frequency was calculated dividing the number of Ki67 positive cells in the crypt by the total number of crypt enterocytes. Each point represents the frequency of positive cells in intestinal biopsies from one single subject cultured with medium alone or with anti-IL-15 antibody. (**C)** TNF-α and (**D**) IFN-γ were measured by ELISA in the organ culture supernatants of intestinal mucosa. Horizontal bars are the mean values. The paired Student t test was applied to calculate the statistical significant differences.

We next evaluated the role of IL-15 on IBD mucosal inflammation. Functional studies were performed on 5 CD and 7 UC intestinal biopsies cultured with antibody neutralizing IL-15. After 24 hours of *in vitro* treatment, the number of lamina propria CD25+ cells was reduced in both CD and UC patients (mean ± SEM: 45 ± 9.76 versus 80 ± 39.35 in CD, p = 0.06; 67.57 ± 21.05 versus 106.9 ± 34.37 in UC, p = 0.05), **Fig 7A**. The reduction of the number of CD25+ cells upon blocking of IL-15 was more evident in the biopsies of the overall IBD patients, p = 0.005). Similarly, we found a reduced percentage of proliferating crypt enterocytes (Ki67+ cells) in the anti-IL-15 cultured biopsies of both CD and UC patients with a statistically significant decrease in the overall group of IBD patients (27.98 ± 10.65% in IL-15 cultured mucosa, versus 33.69 ± 12.35% in medium cultured mucosa, p = 0.04)**, Fig 7B.**

To further evaluate the immune modulating effect of blocking IL-15 in IBD intestinal inflammation, we measured by ELISA the levels of IFN-γ and TNF-α secreted in the organ culture supernatants. The amount of TNF-α was found reduced in organ cultures of both CD and UC patients, with a statistically significant reduction in CD patients (1269 ± 301.4 pg/mL in biopsies culture with medium alone versus 1022±193.4 pg/mL, in biopsies cultured with anti-IL-15, p = 0.02), **Fig 7C**. By contrast, the level of IFN-γ in organ culture supernatants was low in the great majority of patients, in agreement with IFN-γ production profile of cultured intestinal cells, and resulted slighted reduced upon treatment with IL-15 blocking antibody, **Fig 7D**.

## Discussion

We have investigated the proinflammatory cytokine profile, through an *ex vivo* analysis, in the intestinal mucosa of paediatric subjects with Crohn’s disease and ulcerative colitis, the two main forms of inflammatory bowel diseases. The cytokine production was evaluated in different intestinal cell subsets, such as enterocytes, dendritic cells and T cells, by an experimental approach that combined flow cytometry and ELISA done on freshly isolated cells from IBD patients and non-IBD children. Furthermore, we also investigated the role of IL-15 in intestinal inflammation by a brief in vitro treatment of IBD intestinal biopsies with neutralizing antibodies. We found a marked increase of cells expressing IL-15 in both CD and UC in comparison to healthy mucosa. The density of cells producing TNF-α or IFN-γ was also enhanced in IBD compared to control gut, though in a lesser extent compared to IL-15+ cells. Immunohistochemistry analysis further demonstrated a marked expression of IL-15 on the epithelium surface in the intestinal mucosa of IBD patients. Blockage of IL-15 by a brief treatment of mucosal explants with a specific IL-15 antibody significantly reduced mucosal inflammation, thus suggesting a potential role of IL-15 in paediatric IBD inflammatory cascade.

By using specific cell markers and a multiparametric flow cytometry analysis, we revealed that both immune and nonimmune cells were source of proinflammatory cytokines in paediatric IBD intestine. EpCam+ enterocytes expressing IL-15, HLA class I and TNF-α, as well as CD11c+ dendritic cells co-expressing the activation marker HLA-DR and producing TNF-α and INF-γ, were more frequent in IBD compared to non-IBD control biopsies. Interestingly, the enhanced frequency of cytokine producing cells was observed in gut samples at basal condition, most likely induced by an *in vivo* intestinal microenvironment stimuli. Unexpectedly, no marked differences were observed in the frequency of T cell secreting TNF-α and INF-γ among the three groups. It is known that there is an enhanced expression of proinflammatory cytokines in gut mucosa from both UC (mostly Th2 cytokines, IL-5, IL-9, IL-13) and CD (mostly Th1 cytokines: IFN-γ, TNF-α), [6] and it has been postulated these cytokines are directly responsible of tissue injury [6, 40, 41]. Most studies have investigated the pathological events occurring in inflamed areas of intestinal mucosa in adults with IBD, however, it is unclear the status of cells activation and the cytokine profile in unaffected IBD tissues. Therefore, we believe that an analysis in uninflamed areas represents a relevant approach to describe the phenotype of proinflammatory cytokine producing cells in a prior condition of inflammation. Indeed, few studies have described high levels of proinflammatory cytokines in the unaffected areas of IBD intestine, suggesting an abnormal immune activation also in these areas [42, 43]. In particular, Leon et al found that several cytokines, such as IFN-γ, TNF-α, IL-6, IL-15, IL-18 and IL-23, were increased in both affected and unaffected areas from IBD gut specimens compared to healthy controls, measured by ELISA and mRNA expression [43]. Collectively, our finding in paediatric patients are in agreement with an increased expression of both IFN-γ and TNF-α in IBD intestinal mucosa.

To the best of our knowledge, this is the first study that investigates the profile of cytokine producing cells in the gut of paediatric IBD with an *ex vivo* flow cytometry and ELISA approach. There are only two previous studies that analyzed in children the expression profile of proinflammatory and antinflammatory cytokines with a PCR based approach [44, 45] or through immunohistochemistry [45]. Verdier et al [44] observed a differential Th1 and Th17 cytokine expression patterns, depending on the localization of the lesions along the intestine and on the specific pathology of children affected by IBD [44]. Weidlich and coworkers, instead, analysed by PCR and immunohistochemistry the expression of anti-inflammatory IL-37, and they found an increased IL-37 protein expression in submucosal lymphoid cells in young patients with CD and UC that correlated with histological severity score of inflammation [45].

It has been documented that in IBD patients, the intestinal homeostasis system is disrupted and an innate immune response occurs at the level of the enterocytes barrier [46], with tight junction weakening as well as oxidative and immune stress, and inhibition of homeostatic signals secretion [47]. Recent data have highlighted the role of IL-15 as a key player in IBD mucosa lesions, in similar extent to TNF-α and INF-γ [20]. In this study, we demonstrated that there is a higher number of cells producing IL-15 in both CD and UC paediatric intestine and an elevated expression of IL-15 on the epithelial layer of IBD intestinal mucosa. Accordingly, the treatment of IBD intestinal biopsies with anti-IL-15 blocking antibody reduced inflammation by decreasing either the activation of mononuclear cells in the lamina propria and the proliferation of crypt enterocytes. Moreover, the anti-IL-15 antibody determined a reduction of the TNF-α levels in the organ culture supernatants. Interestingly, a similar experimental approach has been used to assess the pro-inflammatory effect of IL-15 in coeliac disease, that suggested the promising use of molecules blocking IL-15 to rescue gut immune homeostasis [48].

IL-15 is a type I cytokine [49, 50] expressed by non-lymphoid cells and it shares the β chain receptor with IL-2 (IL-15/IL-2Rβ), [51]. A large body of findings have indicated that IL-15 is an important mediator of intestinal immune homeostasis, as it exerts an important growth and anti-apoptotic function on intraepithelial TCRγδ+ T cells [52], NK/NKT [53], and cytotoxic CD8+ T cells [54, 55]. As the enterocytes are one of the main source of cells expressing IL-15 in the gut [11], by taking advantage of the specific epithelial cell marker EpCam [56], we monitored the frequency and the phenotype of intestinal epithelial cells in mucosal biopsies of paediatric IBD patients. Both in CD and UC, the enterocytes produced TNF-α and expressed IL-15. An IL-15 overexpression has been reported in several pathological gut conditions, such as celiac disease [17, 35, 32, 57] and chronic inflammatory disorders [19, 20, 40, 58, 59]. In these latter cases, the vast majority of studies investigating the pathogenic role of mucosal IL-15 has looked at the total amount of mRNA transcripts, protein levels by ELISA and western blot in intestinal specimens from adult IBD patients. More specifically, Nishiwaki and co-workers found an increased expression of mRNA of both IL-15 and IL-15Rα receptor in the mucosal tissues of IBD patients, especially in subjects with ulcerative colitis [20]. Interestingly, more recently, Leon et al. showed higher levels of IL-15 in intestinal biopsies from UC and CD patients [40], thus in line with our current findings.

A higher number of EpCam+ cells was found in the gut mucosa of UC patients in comparison to CD and control subjects. The increased frequency of enterocytes in UC mucosa could be due to intestinal epithelium regeneration, as reported in UC patients [60]. Indeed, our results are in accordance to previous studies suggesting that the intestinal epithelium has a strategic role in gut inflammation, it is not only a protective physical barrier to luminal microbiota, but it actively contributes to the activation and proliferation of different cellular components of mucosal immune systems [10].

Within the gut mucosa, DC have the principal role to guarantee the tissue integrity, the protection from pathogens, as well as the tolerance to dietary components [61]. In IBD, DC recruited at the lamina propria have an activated phenotype, they capture and present mucosal antigens to proinflammatory T lymphocytes that secrete cytokines involved in tissue injury and in the loss of immune tolerance [62]. In the gut mucosa of paediatric IBD patients the myeloid DC, identified as CD11c positive cells, had a proinflammatory phenotype, indeed they expressed HLA-DR and produced TNF-α and INF-γ. Of note, we did not find a myeloid DC subset expansion in both CD and UC mucosa. One possible explanation is that we analyzed the phenotype and cytokine profile of myeloid DC taken from uninflamed mucosal areas and in basal condition. The peculiar changes in gut bacterial profiles associated with these diseases, in contrast to the “healthy microbiota” presented in controls, may trigger the DC activation observed in paediatric IBD mucosa [63].

As T lymphocytes are key player in the IBD inflammatory process, by the *ex vivo* analytical approach we looked at the cytokine production profile of T lymphocyte infiltrates in paediatric IBD mucosa. Similarly, to the DC findings, we did not observe an enhanced frequency of CD3+ T cells in IBD compared to healthy samples. By contrast, we found a slight increase of T cells from uninflamed areas of IBD gut spontaneously producing TNF-α and INF-γ. Quite unexpectedly, after a strong stimulation, as induced by PMA/ionomycin molecules, a higher number of activated T cells producing TNF-α and INF-γ was found in controls compared to IBD patients. These data, in evident contrast with the baseline findings, markedly highlighted that proinflammatory T cells are also present in healthy mucosa and they are prone to release TNF-α and/or INF-γ upon strong intestinal stimuli, which overcome the regulatory pathways occurring in healthy mucosa. Therefore, the presence of regulatory T cells controlling the immune responses in healthy gut, or alternatively the intestinal microenvironment favoring the dysbiosis and the inflammatory reaction in IBD, could explain our results.

However, it is well known that the *in vitro* secretion of T cell cytokines can be influenced by the different production profile and may depend on the specific experimental conditions, especially on the mitogens chosen for T cell activation [6, 64]. The influence of the specific stimuli in determining the cytokine profile of intestinal cells was further confirmed, when we measured the levels of TNF-α and INF-γ secreted upon PHA incubation, which is a stimulus less powerful than PMA/ionomycin for the Th1 cytokine secretion [65, 66]. A higher production of these two cytokines was detected both in CD and UC cells, but not in healthy controls. The latest results re-marked a dysregulated activation of mucosal cells related to the specific local stimuli, that occurs in IBD patients.

In conclusion, we demonstrated that both immune competent cells, such as T lymphocytes and dendritic cells, as well as nonimmune cells, such as enterocytes, have a proinflammatory phenotype in intestinal mucosa of paediatric patients with IBD. Our study underlines the relevance of gut epithelial cells as one of the central mediators of mucosal inflammation in IBD. It is becoming clearer that the enterocytes have a role in intestinal mucosa much more active than it has been considered so far. Taking into account that intestinal epithelium cells represent a central node of mucosal cell networks, and that their dysfunction has been related with IBD pathogenesis, these cells could be a new therapeutic target for IBD. Indeed, many studied reported that epithelial cells respond to TNF-α [67], and they are a target of TNF-α inhibitors [68, 69]. Additionally, we found a prominent production of IL-15 in IBD intestinal mucosa, and a reduced inflammation after the blockage of IL-15. Therefore, our data support the hypothesis that monoclonal anti-IL-15 antibodies or pharmacologic agents, that can selectively block IL-15 signal transduction pathways, could be considered as an alternative biological therapy [70], although more functional experiments are necessary to confirm this hypothesis. Moreover, further studies are needed to improve the knowledge of the role of epithelial cells in IBD pathogenesis, as inflammatory nonimmune cells, in order to better state the possible efficacy of therapeutic opportunities targeting epithelial cells in IBD.

## Supporting information

S1 Fig Cytokine production profile after stimulation with bacterial antigen by intestinal myeloid dendritic cells in paediatric IBD. Intestinal CD11c+ dendritic cells (DC) expressing the HLA DR activation marker and producing cytokines were analyzed from intestinal mucosa of CD and UC patients, and from non-IBD controls (HC), by flow cytometry, either at basal condition, or after 2 hours of incubation with *E*. *coli* particles. The frequencies of CD11c+ cells expressing HLA DR and producing TNF-α (**A**), or IFN-γ (**B**) before and after the bacterial pretreatment are shown. Each point represents the percentage of positive cells from one single subjectof each disease/control group, and horizontal bars are the median values. The Wilcoxon test was applied to evaluate statistical significant differences among the two conditions for each group.
(TIF)

## Abbreviations

BSA: bovine serum albumin
CD: Crohn’s disease
DC: dendritic cells
ELISA: enzyme linked immunosorbent assay
EpCam: epithelial cell adhesion molecule
FMOC: fluorescence-minus-one control
HC: non-IBD healthy controls
HLA: human leukocyte antigen
IBD: inflammatory bowel disease
IFN-γ: interferon-γ
IL-12: interleukin-12
IL-17: interleukin-17
PBS: phosphate buffered saline
PHA: phytohaemagglutinin
PPRs: pathogen recognition receptors
TNF-α: tumor necrosis factor-α
UC: ulcerative colitis
