# Supplementary figures and images for: Cytokine production profile in intestinal mucosa of paediatric inflammatory bowel disease
Source: PLoS One. 2017 Aug 10;12(8):e0182313. doi: 10.1371/journal.pone.0182313 (PMC5552230; doi:10.1371/journal.pone.0182313)

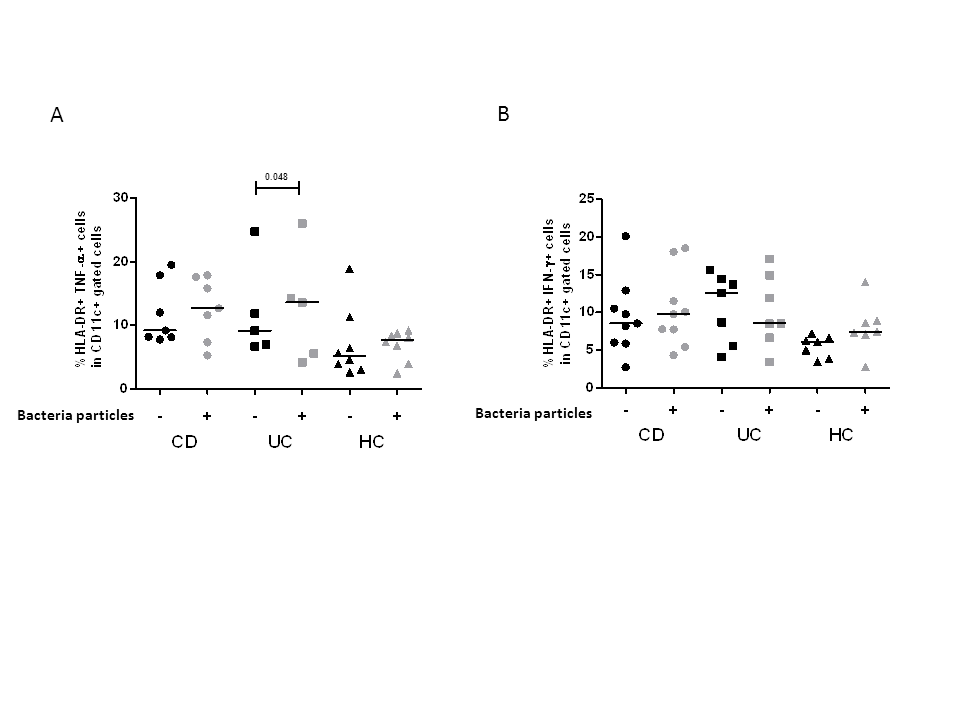

Supplement: S1 Fig — Intestinal CD11c+ dendritic cells (DC) expressing the HLA DR activation marker and producing cytokines were analyzed from intestinal mucosa of CD and UC patients, and from non-IBD controls (HC), by flow cytometry, either at basal condition, or after 2 hours of incubation with E. coli particles. The frequencies of CD11c+ cells expressing HLA DR and producing TNF-α (A), or IFN-γ (B) before and after the bacterial pretreatment are shown. Each point represents the percentage of positive cells from one single subjectof each disease/control group, and horizontal bars are the median values. The Wilcoxon test was applied to evaluate statistical significant differences among the two conditions for each group. (TIF) [file pone.0182313.s001.tif]
